# Assessment of Ki67 and uPA/PAI-1 expression in intermediate-risk early stage breast cancers

**DOI:** 10.1186/s12885-017-3648-z

**Published:** 2017-09-27

**Authors:** Elise Deluche, Laurence Venat-Bouvet, Sophie Leobon, Veronique Fermeaux, Joelle Mollard, Nadira Saidi, Isabelle Jammet, Yves Aubard, Nicole Tubiana-Mathieu

**Affiliations:** 1 Department of Medical Oncology, University Hospital, 2 avenue Martin Luther King, F-87042 Limoges, France; 2 Department of Pathology, University Hospital, F-87042 Limoges, France; 3 Department of Gynaecology, Mother and Child Hospital, F-87042 Limoges, France; 4 Department of Radiotherapy, University Hospital, F-87042 Limoges, France; 5 Department of Senology, Mother and Child Hospital, F-87042 Limoges, France

**Keywords:** uPA/PAI-1, Ki67, Subtypes, Grade II, Breast cancer

## Abstract

**Background:**

The objective of this study was to compare the efficacy of biomarkers in assessing the risk of breast cancer recurrence in patients with node-negative or micrometastatic grade II breast cancer. Specifically, we compared risk assessments based on the St. Gallen clinicopathological criteria, Ki67 expression and urokinase plasminogen activator (uPA)/plasminogen activator inhibitor-1 (PAI-1) expression.

**Methods:**

This retrospective study included 347 patients with breast cancer followed at Limoges University Hospital. The optimal cut-off for high Ki67 expression (Ki67^hi^) was established as 20%. The threshold for uPA and PAI-1 positivity was 3 ng/mg and 14 ng/mg, respectively.

**Results:**

Ki67 expression was lower in uPA/PAI-1-negative than in uPA/PAI-1-positive tumours (227 tumours; *P* = 0.04). The addition of Ki67 status to the St. Gallen criteria resulted in a 28% increase in the rate of identification of high-risk tumours with a potential indication for chemotherapy (*P* < 0.001). When considering uPA/PAI-1 levels together with the St Gallen criteria (including Ki67 expression), the number of cases identified as having a high recurrence risk with a potential indication for adjuvant chemotherapy increased by 20% (P < 0.001). Adjuvant chemotherapy was 9% less likely to be recommended by a multidisciplinary board when using the current criteria compared with using a combination of the St. Gallen criteria and Ki67 and uPA/PAI-1 status (*P* = 0.03).

**Conclusions:**

Taken together, our data show discordance among markers in identifying the risk of recurrence, even though each marker may prove to be independently valid.

## Background

The indication for adjuvant therapy for breast cancer has led to a search for efficient prognostic and predictive biomarkers for patients at greatest risk of local and/or distant recurrence and with a potential indication for chemotherapy (i.e. patients requiring adjuvant therapy). The main objective is to distinguish patients with a low risk of recurrence, for whom little evidence supports the need for chemotherapy, from those with high-risk disease, for whom chemotherapy is clearly justified. The 2007 [1] and 2013 [2] St. Gallen criteria used to define high-risk breast cancer are patient age < 35 years, tumour size >2 cm, tumour grade III, presence of extensive peritumoural vascular invasion, oestrogen receptor (ER) and/or progesterone receptor (PR) negativity, human epidermal growth factor receptor 2 (HER2) overexpression or *HER2* amplification, high Ki67 expression (in grade II tumours) and >3 positive lymph nodes. The presence of any one of these factors is considered sufficient for defining a high risk of recurrence with an indication for adjuvant chemotherapy.

Although grade I and III tumours are biologically and clinically distinct, it is difficult to predict the outcomes of node-negative or micrometastatic (N0) grade II tumours because of their intermediate risk of recurrence [3]. Furthermore, the ultimate benefit of adjuvant chemotherapy for these patients is uncertain. Promising biomarkers used to stratify patients into different risk groups include Ki67 and urokinase plasminogen activator (uPA)/plasminogen activator inhibitor-1 (PAI-1) [4–7]. Reproducible data at a I-B level of evidence (LoE) suggest that Ki67 is a prognostic marker in early stage breast cancer [8], as well as a positive predictive factor for adjuvant chemotherapy [9], especially in patients with luminal B tumours [10]. In addition to the traditional parameters, guidelines recommend using proliferation markers, such as Ki67, to define patient subgroups [2].

The prognostic and predictive abilities of the tumour-associated proteolytic factor uPA, and its inhibitor PAI-1, in patients with N0 disease have been demonstrated at the highest LoE (LoE I-A) [11]. In the Chemo N0 trial, uPA/PAI-1 was identified as a clinically significant risk discriminator in the clinically relevant grade II breast cancer subgroup [12]. Furthermore, in N0 breast cancer, especially grade II tumours, uPA and PAI-1 are predictive markers for the response to cyclophosphamide, methotrexate and 5-fluorouracil (CMF) chemotherapy (LoE I-A) [13]. Based on the high LoE, using uPA/PAI-1 status as an indicator for adjuvant chemotherapy for ER/PR-positive, HER2-negative (node-negative) breast cancer has been recommended by international guidelines [11]. uPA/PAI-1 expression distinguishes high-risk patients expected to receive a major benefit from chemotherapy from low-risk patients with a low probability of benefitting from chemotherapy.

The objective of this study was to assess the predictive value of the St. Gallen clinicopathological criteria, Ki67 status and uPA/PAI-1 status in patients with N0 grade II breast cancer.

## Methods

This retrospective study was performed from December 2007 to October 2015 at Limoges University Hospital, France.

Patients diagnosed with breast cancer, and with complete data available regarding their surgically resected tumours, including tumour Ki67, ER, PR, HER2 and uPA/PAI-1 status, were eligible for this study. Exclusion criteria were missing data for any of the abovementioned tumour markers, macroscopic lymph node involvement, previous breast cancer, initial metastatic breast cancer or prior neoadjuvant chemotherapy. Patients with microscopic lymph node involvement or isolated cells were included, because these factors do not influence the decision to perform adjuvant chemotherapy [14]. Clinical data were collected in accordance with French bioethics laws regarding patient information and consent. Patient consent to the use of their data and biological material was sought prior to the commencement of medical care.

Clinicopathological subtypes, as well as the risk of tumour recurrence, were defined according to the St. Gallen criteria [1, 2]. All Ki67 staining was performed by the same pathological laboratory using the MIB1 monoclonal antibody (1:80 dilution; Dako, Glostrup, Denmark); the largest tumour area, including the most proliferative zone, was assessed. The Ki67 score was calculated as the percentage of immunostained cells. The optimal cut-off for a high versus low Ki67 score was defined as 20% (i.e. ≥ 20% staining was defined as Ki67^hi^), according to the 2015 recommendations [15].

Quantitative evaluation of uPA and PAI-1 concentrations was performed at the Biological Oncology Laboratory (Marseille, France) using the commercially available FEMTELLE® enzyme-linked immunosorbent assay. Positive uPA expression was defined as >3 ng/mg protein, and positive PAI-1 expression as >14 ng/mg protein. uPA/PAI-1 positivity, defined as an elevation of at least one of these markers [16], has been used to identify high-risk tumours [11]. The uPA/PAI-1 markers can be used independently of the St. Gallen criteria [17].

Our multidisciplinary breast cancer team set up the treatment program for the patients. Regional recommendations were based on the St. Gallen criteria and uPA/PAI-1 status. According to a high LoE (I-A), uPA and/or PAI-1 are the preferred markers used to indicate adjuvant chemotherapy for N0 grade II, ER/PR-positive tumours.

### Statistical analyses

Nominal variables were compared among groups using the chi-square test or Fisher’s exact test, as appropriate. Means were compared using the nonparametric Mann-Whitney U-test for continuous variables, and the Kruskal-Wallis test was used for comparisons of ordinal variables among more than two groups. A *P* value <0.05 was considered to indicate statistical significance. Statistical analyses were performed using STATVIEW® software (SAS Institute Inc., Cary, NC, USA).

## Results

### Clinical and histological characteristics

We screened 2300 patients with breast cancer treated from December 2007 to October 2015. Application of our study inclusion criteria resulted in a final cohort of 347 patients, selected primarily because they had available uPA/PAI-1 data. All tumours were evaluated (Tables 1–3), and the results from grade II tumours were used specifically, because the use of uPA/PAI-1 expression as a recurrence marker has been validated in these tumours only.

**Table 1:** Patient and tumour characteristics according to Ki67 expression or uPA/PAI-1 status (*n* = 347)

			Age, Years	Menopausal status		Tumour classification				Hormonal status				HER2 status		Clinicopathological subtypes				Histological type					Histological grade		
		n (%)	Median (range)	Premenopausal	Postmenopausal	T1	T2	T3	T4	ER+/PR +	ER+/PR-	ER−/PR+	ER−/PR-	Negative	Positive	Luminal A	Luminal B HER2-negative	Luminal B HER2-positive	HER2-positive	Triple-negative	Invasive ductal carcinoma	Invasive ductal + lobular carcinoma	Invasive lobular carcinoma	Other	I	II	III
Total		347	62 (33–87)	85 (25)	262 (75)	231 (66)	108 (31)	6 (2)	2 (1)	260 (75)	62 (18)	0 (0)	25 (7)	333 (96)	14 (4)	190 (55)	120 (34)	12 (3)	2 (1)	23 (7)	245 (71)	12 (3)	68 (20)	22 (6)	74 (21)	227 (66)	46 (13)
Ki67 status	Low	250 (72)	61.5 (33–87)	60 (24)	190 (76)	178 (71)	65 (26)	5 (2)	2 (1)	196 (78)	49 (20)	0 (0)	5 (2)	242 (97)	8 (3)	189 (76)	48 (19)	8 (3)	0 (0)	5 (2)	160 (64)	10 (4)	59 (24)	21 (9)	71 (28)	170 (68)	9 (4)
	High	97 (28)	61 (36–85)	25 (25)	72 (75)	53 (55)	43 (44)	1 (1)	0 (0)	64 (66)	13 (13)	0 (0)	20 (21)	91 (94)	6 (6)	1 (1)	72 (74)	4 (4)	2 (2)	18 (19)	85 (88)	2 (2)	9 (9)	1 (1)	3 (3)	57 (59)	37 (38)
*P-*value				0.7		0.02				<0.0001				0.1		<0.0001				<0.0001					<0.0001		
uPA/PAI-1 status	Negative	135 (39)	60 (38–87)	40 (30)	95 (70)	79 (59)	53 (39)	2 (1)	1 (1)	99 (74)	30 (22)	0 (0)	6 (4)	131 (97)	4 (3)	77 (57)	49 (36)	3 (2)	1 (1)	5 (4)	86 (64)	8 (6)	32 (24)	9 (7)	36 (27)	85 (63)	14 (10)
	Positive	212 (61)	61 (33–86)	45 (21)	167 (89)	152 (71)	55 (26)	4 (2)	1 (1)	161 (76)	32 (15)	0 (0)	19 (9)	202 (95)	10 (5)	113 (53)	71 (33)	9 (4)	1 (1)	18 (9)	159 (75)	4 (19)	36 (17)	13 (7)	38 (18)	142 (67)	32 (15)
*P-*value				0.07		0.08				0.09				0.5		<0.001				0.1					0.1		

Table 1 summarises the patient and tumour characteristics according to Ki67 and uPA/PAI-1 status. Ki67 expression was considered low in 250 (72%) tumours (median Ki67 level = 10%; range: 1–15%) and high in 97 (28%) tumours (median Ki67 level = 30%; range: 20–80%). uPA/PAI-1 expression was negative in 135 (39%) tumours and positive in 212 (61%) tumours, and was associated with the clinicopathological subtype (*P* < 0.001). The median uPA expression level was comparable between single (uPA) and double-positive tumours (uPA plus PAI-1; Table 2).

**Table 2:** Median uPA/PAI-1 levels in tumour populations

	uPA−/PAI-1+	uPA+/PAI-1+	uPA +/PAI-1-	uPA−/PAI-1-
Number of patients	85	92	35	135
Median level of uPA (ng/mg; min-max)	1.7 (0.19–2.9)	4.7 (3.0–12.9)	4.7 (3–7.3)	1.2 (0.0–2.9)
Median level of PAI-1 (ng/mg; min-max)	21.1 (14.0–87.8)	22.6 (14.0–130)	9.56 (5.5–13.8)	6.7 (2.9–10.6)

uPA+: uPA ≥3 ng/mg; uPA-: < 3 ng/mg; PAI-1+: ≥14 ng/mg; PAI-1-: <14 ng/mg

Low Ki67 expression was associated with pT1, luminal A, ER- and PR-positive, invasive lobular carcinoma and grade II tumours, while high Ki67 expression was associated with pT2, luminal B, HER2-, ER- and PR-negative (triple-negative), invasive ductal carcinoma and grade III tumours (*P* < 0.05). Negative uPA/PAI-1 expression was associated with luminal A tumours, while positive uPA/PAI-1 expression was associated with luminal B HER2-negative tumours (*P* < 0.001).

Table 3 shows the uPA/PAI-1 and Ki67 levels stratified according to tumour grade. No association was observed between Ki67 and uPA/PAI-1 expression in tumours, irrespective of the histological grade. There were no significant associations between Ki67 and uPA/PAI-1 expression in either grade I (*P* = 0.5) or grade III (*P* = 0.1) tumours. However, in grade II tumours, there was an association between low Ki67 and negative uPA/PAI-1 expression, and between high Ki67 and positive uPA/PAI-1 expression (*P* = 0.04).

**Table 3:** uPA/PAI-1 and Ki67 levels stratified according to tumour grade

GRADE I						
	Positive			Negative		Positive vs. negative uPA/PAI-1
Ki67	uPA+/PAI-1+	uPA +/PAI-1-	uPA−/PAI-1+	uPA−/PAI-1-	Total	*P* value
< 20%	11 (16)	4 (6)	22 (31)	34 (47)	71	0.5
≥ 20%	1 (33)	0 (0)	0 (0)	2 (67)	3	
GRADE II						
	Positive			Negative		Positive vs. negative uPA/PAI-1
Ki67	uPA+/PAI-1+	uPA+/PAI-1-	uPA−/PAI-1+	uPA−/PAI-1-	Total	*P* value
< 20%	42 (25)	15 (9)	43 (25)	70 (41)	170	0.04
≥ 20%	24 (42)	6 (10)	12 (21)	15 (27)	57	
GRADE III						
	Positive			Negative		Positive vs. negative uPA/PAI-1
Ki67	uPA+/PAI-1+	uPA+/PAI-1-	uPA−/PAI-1+	uPA−/PAI-1-	Total	*P* value
< 20%	3 (33)	4 (45)	1 (11)	1 (11)	9	0.1
≥ 20%	11 (30)	6 (16)	7 (19)	13 (35)	37	

uPA+: uPA ≥3 ng/ml; uPA- < 3 ng/ml; PAI-1+: ≥ 14 ng/ml; PAI-1-: <14 ng/ml. Data are reported as *n* (%) unless otherwise stated

### Ki67 levels in patients with N0 grade II tumours

**Fig. 1:**
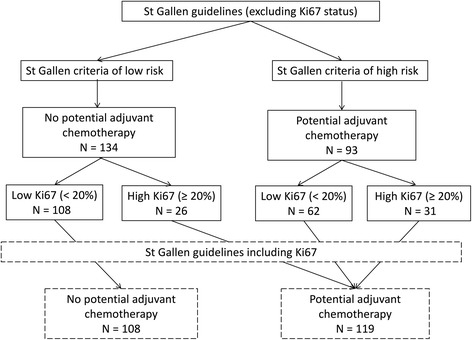
Risk of recurrence and indication for chemotherapy according to the St. Gallen criteria and Ki67 status

The St. Gallen low-risk criteria, excluding the Ki67 status, were satisfied in 134 cases, who were thus deemed to have no indication for potential adjuvant chemotherapy. Of these 134 cases, 108 had a Ki67^low^ status and 26 a Ki67^hi^ status (Fig. 1).

The St. Gallen high-risk criteria, excluding the Ki67 status, were satisfied in 93 cases, of whom 31 were Ki67^hi^ and 62 Ki67^low^ (Fig. 1). In the Ki67^low^ tumours, the corresponding St. Gallen high-risk criteria were pT2 stage (*n* = 55), and/or the presence of vascular emboli (*n =* 5), and/or HER2-positivity (*n =* 5) or a triple-negative status (*n =* 4).

When the Ki67 status was added to the St. Gallen criteria, 119 cases were considered to have a high risk of recurrence. This effectively increased the percentage of cases with a potential indication for adjuvant chemotherapy by 28% compared with that using clinicopathological parameters alone (*P* < 0.001).

### The uPA/PAI-1 status in patients with N0 grade II tumours

**Fig. 2:**
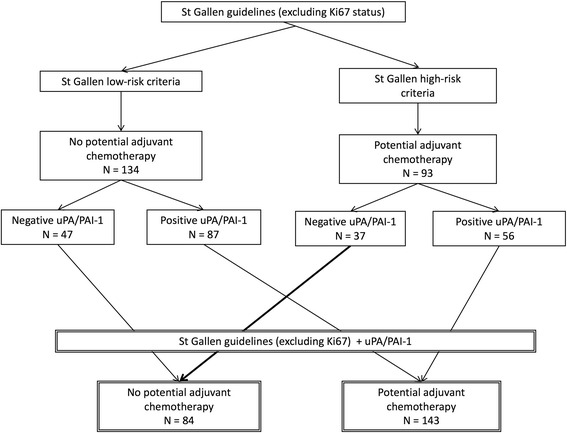
Risk of recurrence and indication for chemotherapy according to the St. Gallen criteria and urokinase plasminogen activator (uPA)/plasminogen activator inhibitor-1 (PAI-1) status

Of the 134 cases who satisfied the St. Gallen low-risk criteria (excluding the Ki67 status), uPA/PAI-1 expression was negative in 47 cases and positive in 87; thus, a need for adjuvant chemotherapy was indicated in the latter group (Fig. 2). Of the 93 cases who satisfied the St. Gallen high-risk criteria (excluding the Ki67 status), 56 were positive and 37 were negative for uPA/PAI-1 expression (Fig. 2).

### Contribution of Ki67 and uPA/PAI-1 status to risk assessment in patients with N0 grade II tumours

**Fig. 3:**
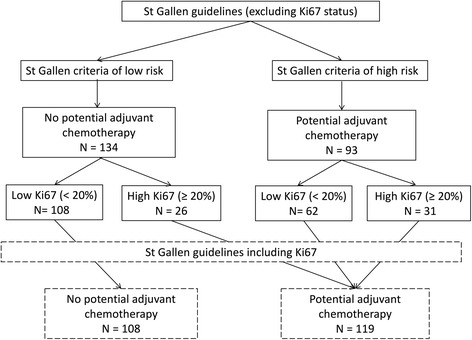
Risk of recurrence and indication for chemotherapy according to the St. Gallen criteria, Ki67 status and uPA/PAI-1 status

The St. Gallen low-risk criteria, inclusive of the Ki67 status, were satisfied in 108 cases. Among these cases, Ki67 and uPA/PAI-1 status was concordant in 41 cases (i.e. Ki67^low^ and uPA/PAI-1 negative) and discordant in 67 cases (i.e. Ki67^low^ and uPA/PAI-1 positive) (Fig. 3).

The St. Gallen high-risk criteria, inclusive of the Ki67 status, were satisfied in 119 cases, with concordant Ki67 and uPA/PAI-1 expression observed in 70 cases. Of these concordant cases, 42 were Ki67^low^ (uPA/PAI-1 negative) and 28 were Ki67^hi^ (uPA/PAI-1 positive). Of the 49 cases with discordant Ki67 and uPA/PAI-1 expression, 34 were Ki67^low^ (uPA/PAI-1 positive) and 15 were Ki67^hi^ (uPA/PAI-1 negative).

Inclusion of both the uPA/PAI-1 and Ki67 criteria increased the rate of identification of tumours with a high risk of recurrence (and thus with a potential indication for adjuvant chemotherapy) by 20% (*P* < 0.001) compared with using the St. Gallen criteria alone.

### The uPA/PAI-1 status, St. Gallen criteria and multidisciplinary board decision to perform adjuvant chemotherapy in patients with N0 grade II breast cancer

Of the 84 cases considered to be at a low risk of recurrence (as defined by the uPA/PAI-1 status), 43 satisfied the St. Gallen high-risk criteria, with 11 (25%) found to be Ki67^hi^. The St. Gallen high-risk criterion in these cases was a tumour size >2 cm. The multidisciplinary board proposed chemotherapy for only five cases (1%), in whom a grade III tumour was identified in the biopsy materials but not in the surgical specimens.

Of the 143 cases considered to be at a high risk of recurrence (as defined by the uPA/PAI-1 status), 67 satisfied the St Gallen low-risk criteria. Our multidisciplinary board decided not to recommend chemotherapy in 18 of these cases because of problems in interpreting the uPA/PAI-1 data (*n =* 2), comorbidities or old age (*n =* 10), or St. Gallen low-risk criteria discordant with UPA/PAI-1 positivity (*n* = 6).

The median follow-up for the entire population was 33 months (range: 1–82 months), which was too short to assess patient outcomes.

## Discussion

To our knowledge, this is the first study to compare recurrence risk as defined by the St. Gallen clinicopathological criteria, Ki67 status and uPA/PAI-1 status in patients with N0 grade II breast cancer.

This report emphasises the utility of the uPA/PAI-1 status and St. Gallen criteria, including the Ki67 status, for identifying tumours at a high risk of recurrence and thus with a potential indication for chemotherapy. Currently, the use of Ki67 and uPA/PAI-1 as biomarkers remains controversial in adjuvant chemotherapy decision-making. The use of Ki67 is supported by the St. Gallen recommendations [2, 15], and the use of uPA/PAI-1 has been validated by both The American Society of Clinical Oncology (ASCO) [11] and The National Institute of Cancer [18].

Ki67 expression can be used to stratify patients with N0 grade II tumours into two distinct subgroups according to outcome [4, 19]. For example, Aleskandarany et al. reported that ≥10% Ki67 positivity was a prognostic factor for progression-free survival (*P* < 0.001) and overall survival (*P* < 0.001) [4]. Adjuvant chemotherapy for luminal B, HER2-negative, N+ tumours was also found to be beneficial in Ki67^hi^ tumours [20].

In the present study, Ki67 analyses were performed in accordance with the St. Gallen criteria [15], using a threshold of 20% to define those at risk of relapse; however, this cut-off is not optimal given Ki67’s continuous distribution. In this context, the percentage of Ki67^hi^ tumours (28%) was almost as high as that reported previously (32%) in a large study conducted by Penault-Lorca et al. [21]. By using Ki67 in combination with the other St. Gallen criteria it was possible to distinguish tumours according to their risk of recurrence, which improved the rate of identification of high-risk tumours by 19%.

The impact of uPA-PAI-1 staining was previously demonstrated in a study by the European Organization for Research and Treatment of Cancer [17], and in a prospective clinical therapy trial, Chemo N0 [13]. Recently, a long-term 10-year follow-up of Chemo N0 participants confirmed the prognostic value of uPA/PAI-1 in N0 breast cancer, especially in grade II tumours (hazard ratio, 1.94; 95% confidence interval: 1.16–3.24; *P* = 0.01) [12]. For this reason, ASCO recently recommended using uPA/PAI-1 expression to guide decision-making for adjuvant systemic therapy in patients diagnosed with ER/PR-positive, HER2-negative, N0 breast cancer [11]. However, the chemotherapy regimen used in the chemo N0 trial was CMF, which is not routinely used in our practice and is less effective than current treatments with anthracyclines [22]. A prospective study comparing cyclophosphamide, 5-fluorouracil and an anthracycline (FEC) with FEC plus docetaxel has yet to be published [23].

We considered that 63% of the tumours evaluated in this study had a high risk of recurrence based on their uPA/PAI-1 level, which potentially increases the indications for adjuvant chemotherapy. These data agree with those of Saadoun et al., in that incorporation of uPA/PAI-1 with other makers increased the indications for adjuvant chemotherapy [24].

Even though measurement of uPA/PAI-1 levels provides good external quality control [13], some technical and organisational difficulties exist. An assessment of uPA/PAI-1 expression was not performed in 45% of the 2300 patients during our routine examinations because of delayed delivery (> 1 h), the initial size of the tumour (< 1 cm) and/or the need for frozen tissue. Nevertheless, uPA/PAI-1 remains a feasible, low-cost test.

In the second part of this study, we evaluated the definition of recurrence risk according to uPA/PAI-1 expression combined with the St. Gallen criteria, inclusive of Ki67. Previous studies have shown that the use of uPA/PAI-1 combined with a clinicopathological parameter, such as vascular invasion, is extremely helpful for yielding prognostic data [24]. We identified a correlation between Ki67 and uPA/PAI-1 status in grade II tumours, in agreement with Kolben et al. [25], although we lack an explanation for this correlation.

The efficacy of uPA-PAI-1 testing compared with molecular signatures for recurrence risk assessment is not clear. The West German Study Group-Plan B trial is the first study to evaluate the correlation between the Oncotype Dx® recurrence score and uPA/PAI-1 status, which were prospectively compared as risk indicators in a phase III trial setting in patients with early stage breast cancer. A high-risk status, as determined by the recurrence score, was also found to be predictive of a high risk of recurrence using uPA/PAI-1 measurements, while the reverse was not found to be true [26].

This study also evaluated adjuvant chemotherapy decision-making by a multidisciplinary board at our institution. This board currently defines a low risk of recurrence based on the St. Gallen low-risk criteria (including Ki67 status) in addition to a negative uPA/PAI-1 status. If either uPA or PAI-1 expression is elevated, the tumour is considered to be at a high risk of recurrence. Among the study population, 37% of tumours were considered to be at a low risk of recurrence, with no indication for adjuvant chemotherapy. In five cases (6%), the board disagreed with the indication for chemotherapy because of discordance among the uPA/PAI-1 levels, clinicopathological criteria and Ki67 expression. This rate of indication for adjuvant chemotherapy (i.e. 6%) was similar to that in a previous report [27], but lower than the rate of 13% reported by Kolben et al. [25]. However, the cut-off value for Ki67 in those studies was different from that in the current study (10–15% in Kolben et al. and Vénat-Bouvet et al. vs. 20% in our study). In addition, 18 of the 143 tumours (14%) considered at high risk of recurrence did not receive chemotherapy because of comorbidities and/or considerable discordance among prognostic markers.

While the retrospective design of this study was not optimal for drawing conclusions regarding the efficacy of uPA/PAI-1 and Ki67 as markers informing decision-making (regarding adjuvant chemotherapy) by a multidisciplinary board, the data suggest a trend. The other limitation was the variability in the Ki67 staining cut-off value and its impact on the decision to perform adjuvant chemotherapy. Specifically, from 2009 [28] to 2015, the cut-off value has increased from 13.25% to 20–29% [15]. Our results need to be confirmed by a prospective, randomised study to validate uPA/PAI-1 as a risk marker in patients with an uncertain indication for chemotherapy according to the current standard assessments; we are currently awaiting the results of a similar study (NNBC3) [29]. This work highlights the difficulties in evaluating the precise role and salience of individual risk factors in the decision to perform chemotherapy [27]. Treatment decisions were based primarily on the current guidelines but also took other factors into account, such as patient age, comorbidities, HER2 status and patient preference [30].

## Conclusion

Improvements in breast cancer outcomes are attributed mostly to adjuvant chemotherapy. However, the objective of patient care is not only to prevent recurrence, but also to improve the patient’s quality of life; this requires accurate identification of high-risk patients with a clear justification for chemotherapy. Therefore, the main challenge is determining the individual risk of relapse, particularly in patients with grade II breast cancer. The combination of the St Gallen criteria (including the Ki67 status) and uPA/PAI-1 status could provide a better estimation of the relapse risk, thereby avoiding unnecessary adjuvant chemotherapy and improving the quality of life of these patients.

Taken together, our data show potential discordance among the markers used to stratify the risk of recurrence, even when each marker is validated independently. A prospective study is needed to validate the use of a combination of these markers for risk assessment. In the future, genomic analyses may be combined with prognostic markers to better guide decision-making regarding adjuvant systemic therapy in defined patient subsets.

## Abbreviations

ASCO: American Society of Clinical Oncology
CMF: Cyclophosphamide, methotrexate and 5-fluorouracil
ER: Oestrogen receptor
HER2: Human epidermal growth factor receptor 2
LoE: Level of evidence
N0: Node-negative or micrometastatic
PAI-1: Plasminogen activator inhibitor-1
PR: Progesterone receptor
uPA: Urokinase plasminogen activator
